# A low-cost vision system based on the analysis of motor features for recognition and severity rating of Parkinson’s Disease

**DOI:** 10.1186/s12911-019-0987-5

**Published:** 2019-12-12

**Authors:** Domenico Buongiorno, Ilaria Bortone, Giacomo Donato Cascarano, Gianpaolo Francesco Trotta, Antonio Brunetti, Vitoantonio Bevilacqua

**Affiliations:** 1 Department of Electrical and Information Engineering, Polytechnic University of Bari, Bari, Italy; 2 Apulian Bioengineering s.r.l., Via delle Violette 14, Modugno (BA), Italy; 3 Institute of Clinical Physiology, National Research Council, Pisa, Italy; 4 Department of Mechanics, Mathematics and Management, Polytechnic University of Bari, Bari, Italy

**Keywords:** Classification, Artificial neural network, Support vector machine, Feature selection, Parkinson’s disease, Gait analysis, Finger tapping, Foot tapping, MDS-UPDRS, Microsoft kinect v2

## Abstract

**Background:**

Assessment and rating of Parkinson’s Disease (PD) are commonly based on the medical observation of several clinical manifestations, including the analysis of motor activities. In particular, medical specialists refer to the MDS-UPDRS (Movement Disorder Society – sponsored revision of Unified Parkinson’s Disease Rating Scale) that is the most widely used clinical scale for PD rating. However, clinical scales rely on the observation of some subtle motor phenomena that are either difficult to capture with human eyes or could be misclassified. This limitation motivated several researchers to develop intelligent systems based on machine learning algorithms able to automatically recognize the PD. Nevertheless, most of the previous studies investigated the classification between healthy subjects and PD patients without considering the automatic rating of different levels of severity.

**Methods:**

In this context, we implemented a simple and low-cost clinical tool that can extract postural and kinematic features with the Microsoft Kinect v2 sensor in order to classify and rate PD. Thirty participants were enrolled for the purpose of the present study: sixteen PD patients rated according to MDS-UPDRS and fourteen healthy paired subjects. In order to investigate the motor abilities of the upper and lower body, we acquired and analyzed three main motor tasks: (1) gait, (2) finger tapping, and (3) foot tapping. After preliminary feature selection, different classifiers based on Support Vector Machine (SVM) and Artificial Neural Networks (ANN) were trained and evaluated for the best solution.

**Results:**

Concerning the gait analysis, results showed that the ANN classifier performed the best by reaching 89.4% of accuracy with only nine features in diagnosis PD and 95.0% of accuracy with only six features in rating PD severity. Regarding the finger and foot tapping analysis, results showed that an SVM using the extracted features was able to classify healthy subjects versus PD patients with great performances by reaching 87.1% of accuracy. The results of the classification between mild and moderate PD patients indicated that the foot tapping features were the most representative ones to discriminate (81.0% of accuracy).

**Conclusions:**

The results of this study have shown how a low-cost vision-based system can automatically detect subtle phenomena featuring the PD. Our findings suggest that the proposed tool can support medical specialists in the assessment and rating of PD patients in a real clinical scenario.

## Background

Nowadays neurological disorders represent the leading cause of disability [1]. Among neurological disorders, Parkinson’s disease (PD) affects more than six millions of people in the world and is the fastest growing so that the estimated number of PD affected people in the 2040 is 13 million [2]. PD is a neurodegenarative disorder caused by a substantial loss of dopamine in the forebrain. The exhibited signs and symptoms, that can be different for everyone, may include tremor, slowed movement, rigid muscles, impaired posture and balance, loss of automatic movements, speech and writing changes [3, 4]. PD diagnosis is typically made by analyzing motor symptoms with clinical scales, such as the Movement Disorder Society – sponsored revision of Unified Parkinson’s Disease Rating Scale (MDS-UPDRS) [5] and the Hoehn & Yahr (HY) [6].

Although several scientific results support the validity of the MDS-UPDRS for rating, subjectivity and low efficiency are inevitable since most of the diagnostic criteria use descriptive symptoms, which cannot provide a quantified diagnostic basis. In fact, PD early signs may be mild and go unnoticed and symptoms often begin on one side of the body and usually remain worse on that side, even after symptoms begin to affect both sides. With this evidence, the development of computer-assisted diagnosis and computer-expert systems is very important [7, 8], especially when dealing with motor features. Hence, a tool that can help neurologists to objectively quantify small changes in motion performance is needed to have a quantitative assessment of the disease.

## Related works

In the last years, machine learning (ML) techniques have been used and compared for PD classification [9], e.g. Support Vector Machine (SVM), Linear Discriminant Analysis (LDA), Artificial Neural Network (ANN), Decision Tree (DT), Naïve Bayes. Most of the published studies investigate two-group classifications, i.e. PD patients vs healthy subjects of control (HC), with promising results obtained [10]. Few works, indeed, presented multiclass classification among patients at different disease stages [9–11].

Researchers have also applied ML to classify PD patients and HC by extracting features related to motor abilities. The majority of the studies based on the analysis of either the lower limb motor abilities or the upper limb motor abilities are usually focused on a single exercises or a single symptom [12–31]. Different technologies have been exploited to capture the analyzed movements, and the most used are optoelectronic systems, wearable sensors like accelerometers and gyroscopes and camera-based systems [32].

Objective and precise assessments of motor tasks are usually performed using large optoelectronic equipment (e.g., 3D-camera-based systems, instrumented walkways) that require heavy installation and a large space to conduct the experiments [33]. Earlier efforts to develop clinic-based gait assessment tools for patients with PD have appeared in the literature over the past two decades. Muro-de-la-Herran et al. [34] and Tao et al. [35], reviewed the use of wearable sensors, such as accelerometers, gyroscopes, magnetoresistive sensors, flexible goniometers, electromagnetic tracking systems, and force sensors in gait analysis (including both kinematics and kinetics), and reported that they have the potential to play an important role in various clinical applications. Among the different proposed wearable sensors, inertial measurement units (IMU) were widely used, even though there are several key limitations that should be considered when considering the use of wearable IMUs as a clinical-based tool, e.g. the gyroscope-based assessment tools suffer from a drifting effect [36]. Systems that are based on low-cost camera might represent a valid solution to overcome both the high cost and encumbrance of an optoelectronic system and the above reported limitation of the IMU-based system. Since the release of the Microsoft Kinect SDK, the Kinect v2 sensor has been widely utilized for PD-related research. Several projects focused on rehabilitation and they proposed experimental ways of monitoring patients’ activities [36–39]. Most of the cited works carried on comparisons of the Kinect v2 sensor in relation to gold standards, as optoelectronic systems, in order to test and quantify its accuracy.

According to the recent trends in the area of intelligent systems for personalized medicine [40–46], it seems clear that there is the necessity for new, low-cost, and accessible technologies to facilitate in-clinic and at-home assessment of motor alterations throughout the progression of PD [47]. In this context, we have proposed a low-cost camera-based system able to recognize and rate PD patients in a completely non-invasive manner. The main novel contributions respect to the state of the art presented above are:

- differently from already published studies, we also considered the classification of the PD severity;
- we used the MS Kinect v2 system to investigate three motor exercise: gait, finger tapping and foot tapping;
- we evaluated a large set of features extracted from the kinematic data (spatio-temporal parameters, frequency and postural variables);
- differently from previous studies on gait analysis for PD classification [36, 37, 39, 48], we also considered postural oscillations and kinematics of upper body parts (trunk, neck and arms);
- we have developed and compared two classifiers (SVM and ANN) able to assess and rate the movement impairment of PD patients using a specific set of features extracted by the recorded movements.

Our main goal is to design and test a mobile low-cost decision support system (DSS) that can be easily used both in specialized hospitals and at home thus implementing the recent telemedicine paradigms. The system aims then to detected the early symptoms of PD, and to provide a tool able to monitor, assess and rate the disease in a non-invasive manner, since the early stages.

## Materials and methods

### Participants

We recruited thirty elderly participants from a local clinical center (Medica Sud s.r.l., Bari, Italy): 14 healthy subjects (10 male and 4 female, 73.5 ±6.4 years, range 65-82 years) and 16 idiopathic Parkinson patients (13 male and 3 female, 74.9 ±7.6 years, range 63-87 years). The PD patients were examined by a medical doctor and rated according to MDS-UPDRS that considers a scoring with five levels, i.e. normal, slight, mild, moderate and severe. In detail, nine patients have been classified as mild (mean age 67.2 years, SD 9.8, age range 54-81) and seven were rated as moderate (mean age 74.1 years, SD 7.1, age range 63-87). None of the patients was classified as either slight or severe.

### Experimental setups

In this study, we considered three motor exercises that involve both the lower and upper extremities of the body: (1) gait, (2) finger tapping and (3) foot tapping. We designed and tested a specific experimental setup for each exercise. All the setups make use of the Microsoft Kinect v2 One RGB-D camera that acquired both color and depth data at 30 Hz. It is important to remark that a) the finger tapping and the foot tapping were performed the same manner as described in the Subsection III.4 and Subsection III.7 of the UDPRS scale, respectively, whereas b) the gait exercise has been performed on a distance, i.e. 2.5-m meters shorter than the path length considered by the Subsection III.10 (10 meters). The motivation for such choice will be further discussed.

In this study, right and left sides of each participant were independently considered, thus the final dataset about the PD patients is composed of 60 instances.

#### Posture and gait

**Fig. 1:**
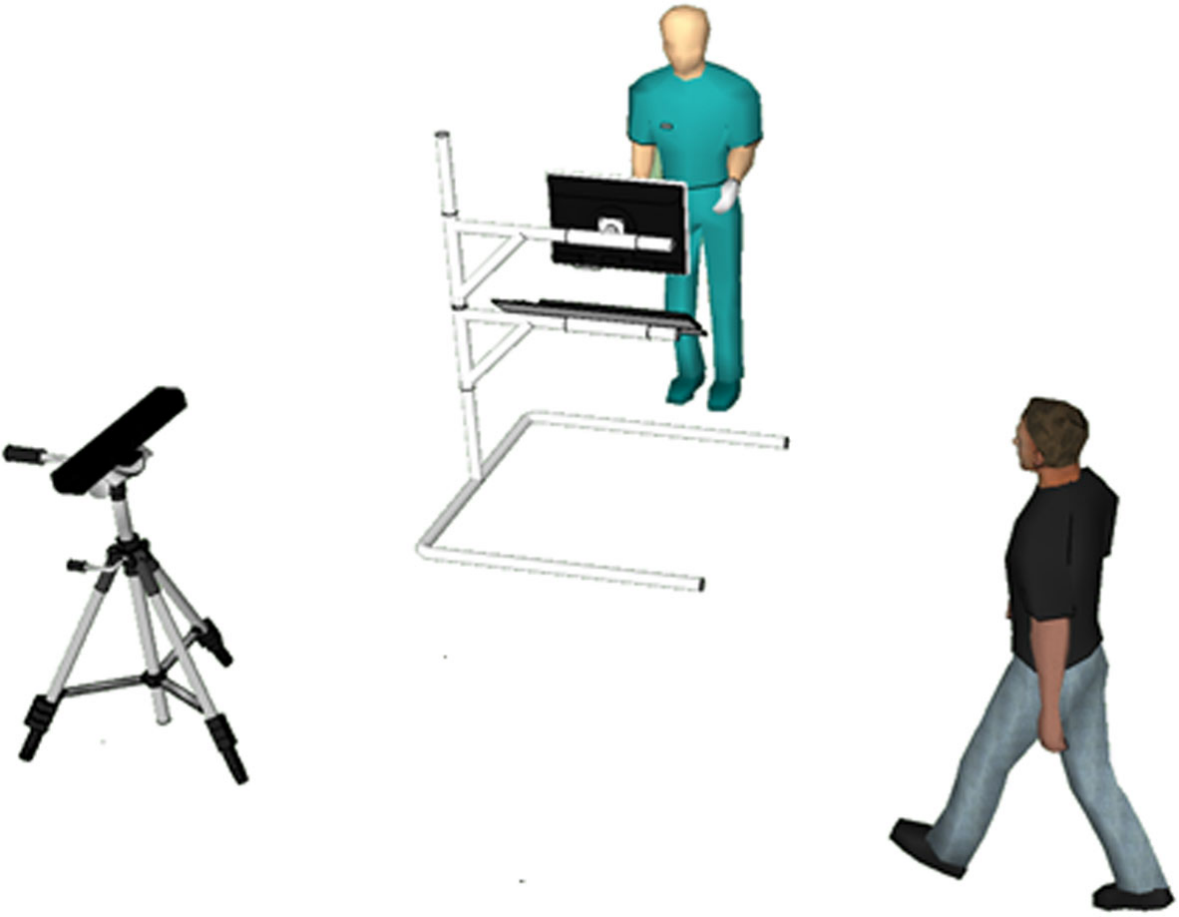
Setup for postural and gait analysis. Representation of the proposed set-up in the clinical center

As the Subsection III.10 of the UDPRS considers, we asked each participant to walk straight towards the camera with the natural normal walking pace (Fig. 1). Participants were asked to repeat the task several times in order to acquire at least one gait cycle (stance phase and swing phase) for each side. The task trial started with the subject standing in a T-pose for one second. Subjects then walked toward the Kinect sensor, which was placed 3.5 m away from the subject’s starting point at a height of 0.75 m. The 3.5 m distance was selected to guarantee that the recorded gait cycle, which began when the subject was about 2.5–3 m from the Kinect, did not include the acceleration/deceleration phases of walking that are anticipated during the initiation or completion of the gait task.

**Fig. 2:**
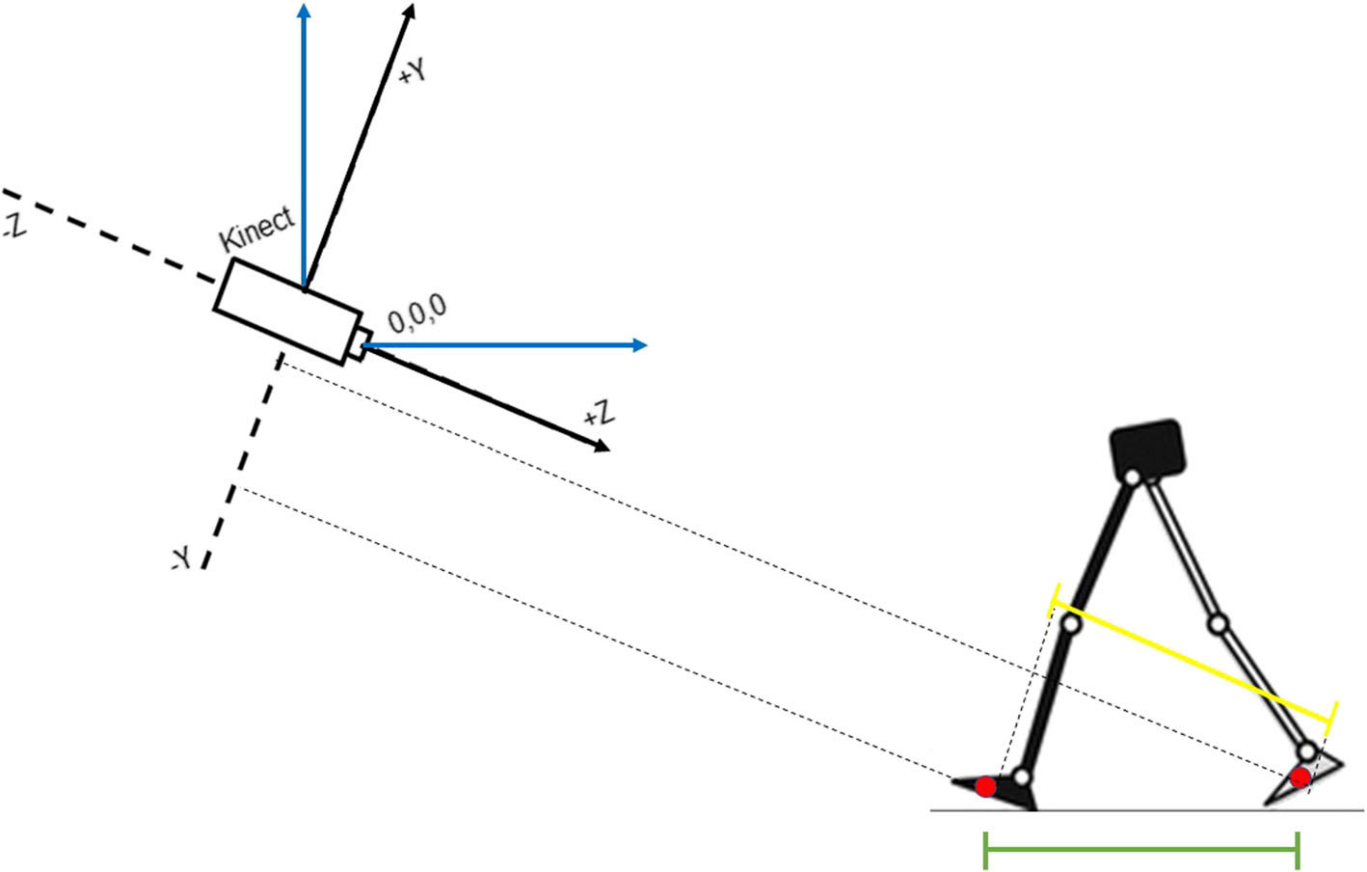
Reference system for postural and gait data acquisition. Schematic Representation of the Global Reference System (the Kinect, black lines) and of the Subject’s Reference System (blue lines)

During the walking, the human skeleton pose has been estimated and recorded by using the Microsoft SDK functions. The resulting human skeleton is represented by 25 nodes, also called control points, in the Kinect’s reference frame known as the skeleton space. Each node represents a specific joint with 3D position information in units of meters. The skeleton space uses a right-handed coordinate system: the Y axis lies in the vertical direction of the image plane, the Z axis extends in depth perpendicularly from the sensor, and the X axis is horizontal in the image plane and orthogonal to the Y and Z axes (Fig. 2). Even though the Subsection III.10 considers a walking distance equal to 10 meters, we used a reduced path length in order to facilitate the skeleton tracking thus reducing the error of the estimated skeleton pose at each frame.

#### Finger tapping

**Fig. 3:**
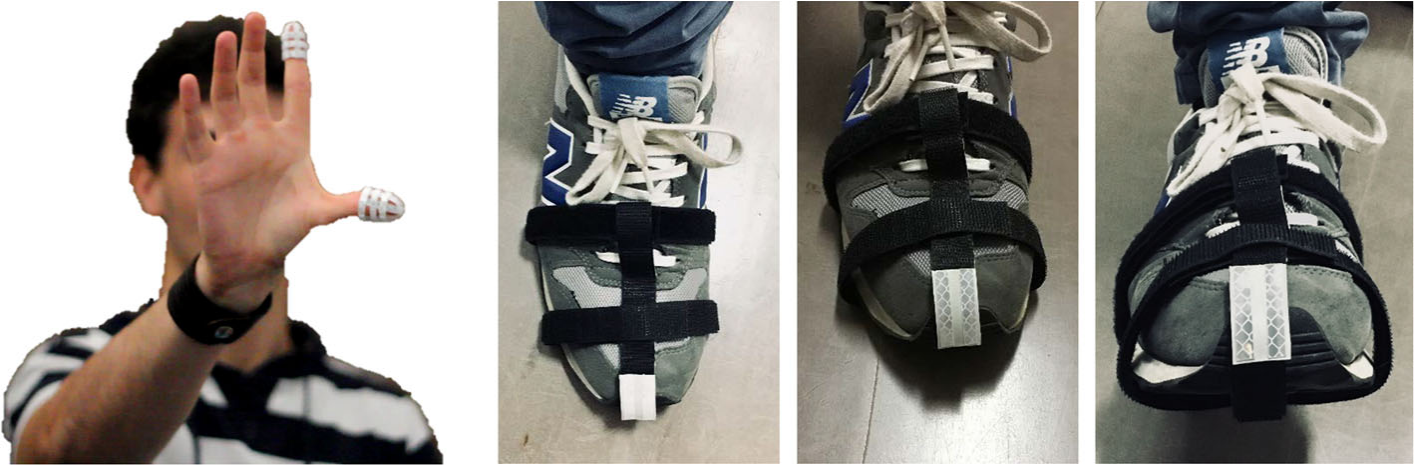
Finger tapping and foot tapping setups. Left image shows a healthy subject wearing the two passive finger markers. The three images reported on the right show the foot of a subject doing the foot tapping exercise while he is wearing a passive marker on the toes

The finger tapping test considers the examination of both hand separately. As the Subsection III.4 of the UDPRS considers, the tested subject is seated in front of the camera and is instructed to tap the index finger on the thumb ten times as quickly and as big as possible. During the task the subject wears two thimbles made of reflective material on both the index finger and thumb (see Fig. 3).

#### Foot tapping

The feet are tested separately. The tested subject sits in a straight-backed chair in front of the camera and has both feet on the floor. He is then instructed to place the heel on the ground in a comfortable position and then tap the toes ten times as big and as fast as possible. A system of stripes with a reflective marker is positioned on the toes (see Fig. 3).

### Movement estimation and feature extraction

For each acquired task, we developed a specific routine able to compute the trajectories of the moving links of the body, i.e. arms, legs, fingers and toes, and to extract a set of hand-crafted features.

#### Gait and postural analysis

As discussed in the experimental setup section, the human skeleton pose has been estimated by using functions of the Microsoft SDK, that automatically computes the 3D position of the 25 landmark points. Given the whole trajectory of each extracted point, three categories of features have been considered:

- temporal features, e.g. duration of gait phases in seconds and in percentage compared to the duration of the gait cycle;
- spatial features, e.g. estimated length, width and velocity of movements, normalized by the height or the lower limb length of the subject according to the specific feature;
- angular features, e.g. the average angle of specific articulations to evaluate the posture of the body and the range of motions of some other skeletal joints.

**Fig. 4:**
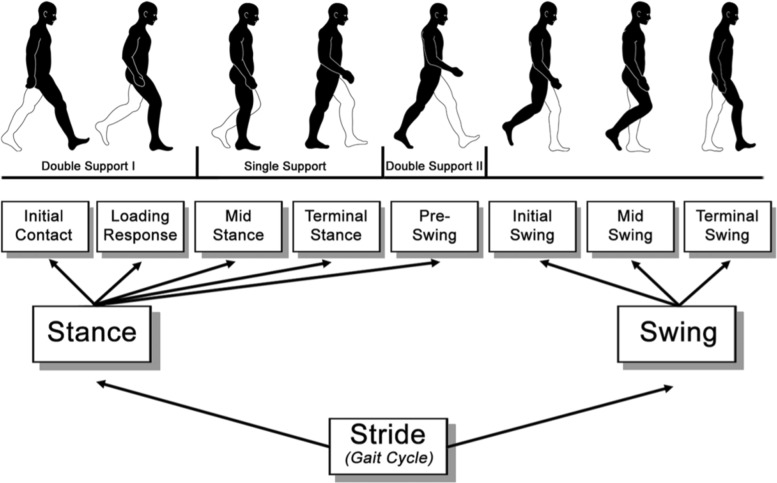
Gait cycle. Breakdown of the gait cycle into phases. Contribution from the work of Stöckel et al. [49]

In detail, we have first segmented each phase of the gait cycle, i.e. Loading Response (LR), Mid-STance (MST), Terminal Stance (TST), Pre-SWing (PSW), Initial SWing (ISW), Mid-SWing (MSW) and Terminal SWing (TSW) (Fig. 4), as proposed by Tupa et al. [38]. Given the exact time of the begin and the end of each gait cycle phase, we then extracted the spatio-temporal features reported into the first 13 rows of the Table 1. Concerning the angular features, that are listed at the bottom of the Table 1, we computed 1) the range of motion (ROM) of the arm swing along the sagittal plane, the average value of the 2) Trunk and the 3) Neck flexion angles along the sagittal plane during the all gait cycle, and the 4) tonic lateral flexion of the trunk along the frontal plane (for more detail about the postural angle definitions please refer to the works of Seah et al. and Barone et al. [50, 51]). The mean and the standard deviation values of both postural and kinematic parameters for all the subjects are summarized in Table 2.

**Table 1:** Postural and gait analysis

Feature	Acronym	Domain	Unit	Selection
Stance Phase	STp	Temporal	%	
Swing Phase	SWp	Temporal	%	
Double Support Phase	DSp	Temporal	%	Case A,B
Stance Time	STt	Temporal	sec	
Swing Time	SWt	Temporal	sec	
Stride Time	STDt	Temporal	sec	Case B
Stride Cadence	STDc	Spatial	#/min	Case A
Stride Length	STDl	Spatial	cm	Case A,B
Step Length	SPl	Spatial	cm	
Step Width	SPw	Spatial	cm	
Stride Velocity	STDv	Spatial	m/sec	Case A
Swing Velocity	SWv	Spatial	m/sec	Case A
Trunk Flexion	TFlex	Angular	degree	Case A,B
Neck Flexion	NFlex	Angular	degree	Case A,B
Pisa Syndrome	PS	Angular	degree	Case A
Arm Swing	ASrom	Angular	degree	Case A,B

Summary of the 16 Features and the Selected Features (9 features for the Case A and 6 features for the Case B) used by the Classification Algorithms

**Table 2:** Postural and gait analysis

Feature	Healthy	PD	Mild PD	Moderate PD
STp	60.1 ±3.3	62.0 ±3.5	61.6 ±2.6	62.5 ±4.4
SWp	39.8 ±3.3	38.0 ±3.5	38.4 ±2.6	37.4 ±4.4
DSp	18.6 ±5.2	22.9 ±5.2	21.8 ±3.7	24.2 ±6.6
STt	0.8 ±0.1	0.8 ±0.1	0.9 ±0.1	0.8 ±0.1
SWt	0.5 ±0.1	0.5 ±0.1	0.6 ±0.1	0.5 ±0.1
STDt	1.3 ±0.1	1.4 ±0.2	1.5 ±0.2	1.3 ±0.2
STDc	45.0 ±5.2	44.9 ±8.3	41.8 ±5.1	48.9 ±10.0
STDl	71.3 ±11.0	56.9 ±15.1	57.3 ±15.3	50.3 ±19.8
SPl	35.8 ±6.2	28.4 ±7.8	28.6 ±7.9	25.1 ±10.0
SPw	8.8 ±2.6	9.7 ±1.9	9.6 ±1.9	10.1 ±2.0
STDv	0.5 ±0.1	0.4 ±0.1	0.4 ±0.1	0.4 ±0.1
SWv	1.2 ±0.3	1 ±0.2	1.0 ±0.2	0.9 ±0.3
TFlex	5.4 ±2.2	5.6 ±2.9	5.6 ±2.9	4.7 ±3.8
NFlex	7.9 ±2.2	8.1 ±2.9	8.0 ±2.9	7.2 ±3.8
PS	0.1 ±1.2	-0.2 ±0.8	-0.2 ±0.8	-0.1 ±0.7
ASrom	16.1 ±7.8	11.0 ±6.3	10.7 ±5.3	10.9 ±8.9

Mean and Standard Deviation of Postural and Kinematic Features during Gait

#### Finger tapping and foot tapping

The acquisition of the finger and foot tapping are based on custom made trackers covered by reflective material. Even though the two tapping are different movements and rely on trackers that have different shapes, a unique algorithm has been used to extract the features related to both exercises. In detail, such procedure considers the extraction of the reflective marker positions from the Microsoft Kinect v2 acquisitions, first, and the computation of all the features related to the acquired movement.

##### Image processing for movement tracking.

The two vision-based acquisition systems use passive reflective markers to track and record the position of the thumb, the index finger and the toes. A routine based on image processing techniques has been developed and employed to 1) recognize the markers in each acquired video frame and 2) compute the 3D position of a centroid point associated to the specific marker. As a first step, the blobs associated with the reflective markers have been segmented using the OpenCV library functions on each infrared image frame as follows:

- Extraction of the pixels associated with the reflective passive markers with a thresholding operation;
- Blurring and thresholding operations in sequence;
- Eroding and dilating operations in sequence;
- Dilating and eroding operations in sequence.

After the post-processing steps explained above, all the found blobs are extracted using an edge detection procedure. Only the blobs having sizes comparable with markers’ size are kept for the next analysis. As final step, the centroid of each blob (only one blob for the foot tapping and two blobs for the finger tapping) is computed. Given the position of the centroid into the image frame, its depth information and the intrinsic parameters of the Kinect V2, we then computed the 3D position of the centroid associated to each tracked marker in the camera reference system. The centroid position has been then considered as the position of the specific fingertip or the foot’s toe.

##### Feature extraction.

**Fig. 5:**
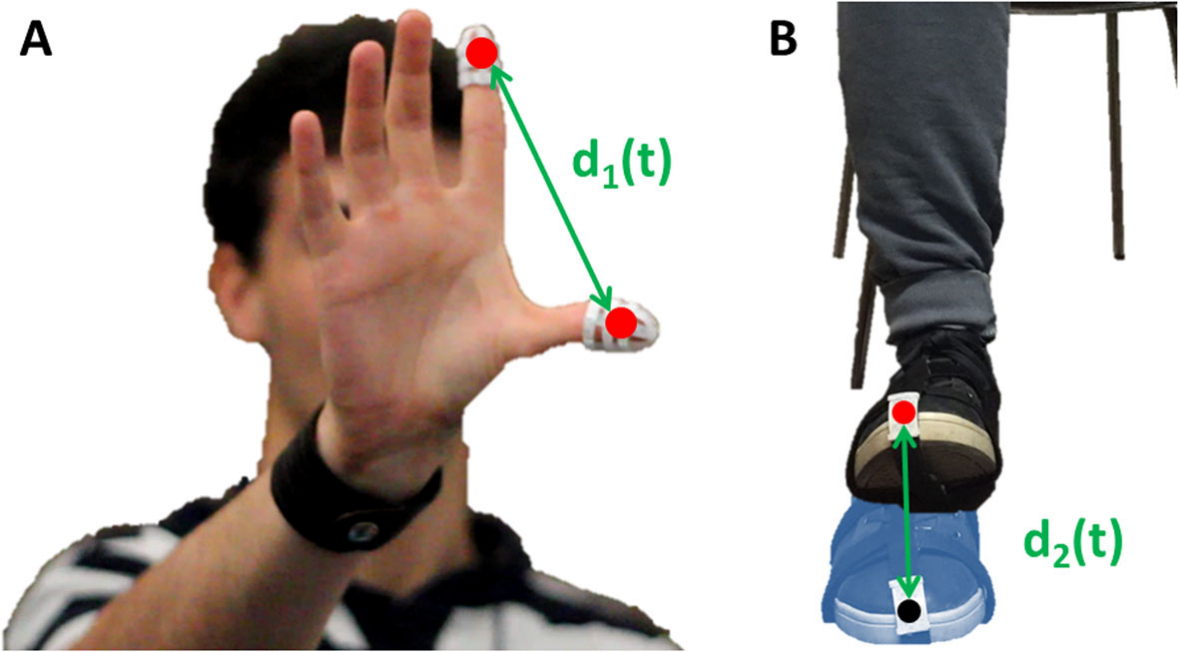
Finger Tapping and Foot Tapping: movement extraction. **a** Finger tapping. The signal d1(t) is the distance between the two centroids (red filled circles) of the passive finger markers. **b** Foot tapping. The signal d2(t) is the distance between the centroid of the toes’ marker and the centroid of the same marker when the toes are completely on the ground
- *d*_2_(*t*) - the distance between the position of the toes’ marker and the position of the same marker when the toes lie on the ground (*Foot Tapping*).

As shown in Fig. 5, the reconstructed marker trajectories have been used to extract the following two signals over time:

- *d*_1_(*t*) - the distance between the index fingers and the thumb markers (*Finger Tapping*);

Both signals have been normalized to make them range in [0,1] since the absolute values of the movement amplitude is not meaningful [5]. Given the entire acquired signal, all the single trials (ten finger tapping and ten foot tapping) have been extracted for each side. We then implemented a simple procedure that automatically extracts the same set of features for both computed signals (d1(t) and d2(t)). The list of features to the time domain, spatial domain and frequency domain follows:

1. **meanTime:** averaged execution time of the single exercise trial;
2. **varTime:** variance of the execution time of the single exercise trial;
3. **meanAmplitude:** averaged space amplitude of the single exercise trial;
4. **varAmplitude:** variance of the space amplitude of the single exercise trial;
5. **tremors:** number of peaks detected during the entire acquisition;
6. **hesitations:** number of amplitude peaks detected in the velocity signal during the entire acquisition;
7. **periodicity:** periodicity of the exercise computed as reported in [52];
8. **AxF:** (amplitude times frequency) the averaged value of the division between the amplitude peak reached in a single exercise trial and the time duration of the trial.

### Classification

In addition to the classification of healthy subjects vs PD patients that has been quite deeply investigated in previous studies [9], in this work we also focused on the classification of PD patients affected by different disease severity. Then, the following two study cases have been conducted:

**Case A: Healthy Subjects versus Parkinson’s Disease patients.** Dataset consists of a total of 30 records, 16 PD patients (53.3%) and 14 older age healthy subjects (46,7%). Right and left sides of each subject were separately considered, then the final dataset is composed of 60 instances.

**Case B: Mild versus Moderate Parkinson’s Disease patients.** Dataset consists of a total of 16 records, 9 mild (56,3%) and 7 moderate (43.7%) PD patients. Right and left sides of each patient were separately considered, then the final dataset is composed of 32 instances.

All the analyses have been conducted following two different strategies based on SVMs and ANNs, which represent state-of-the-art classifiers that have gained popularity within pattern recognition tasks [53–55] and showed promising result in PD classification using kinematic data [9].

Considering the easy tuning of training parameters, SVMs classifiers [56, 57] have been considered to realize a preliminary inspection of the processed data. SVM is a classifier whose goal is to find the best decision hyperplane that separates the training features space. SVMs have high generalization capability because they can be extended to separate a space of non-linear input features [58]. For the purpose of the present work, the training process was based on 5-fold cross-validation and evaluate in sequence the following types of SVM classifiers: (1) linear SVM, (2) quadratic SVM, (3) cubic SVM, (4) Gaussian SVM. We also tested evolutionary approaches, and optimization strategies based on probabilistic graphical models [59, 60], for the design of neural classification architectures [61–64]. In particular, we applied an improved version of the Genetic Algorithm (GA) reported in Bevilacqua et al. [65], where the fitness function maximized by the GA consists in the mean value of accuracy reached by each ANN-based classifier trained with a fixed number of iterations, validated and tested using a random permutation of the dataset instances.

#### Classification based on gait and postural analysis

Concerning the classification based on the features extracted from the gait exercise, for each case (Case A and Case B) we derived two subcases depending on the number of features considered for the classification step (see Table 1). In particular, we ran a correlation based feature selection procedure (*Weka* - Attribute Evaluator: *CfsSubsetEval* - Search Method: *BestFirst*) that has individuated 9 features out of 16 for the Case A and 6 features out of 16 for the Case B:

- Subcase A.1: all 16 features;
- Subcase A.2: 9 selected features out of 16;
- Subcase B.1: all 16 features;
- Subcase B.2: 6 selected features out of 16.

#### Classification based on finger tapping and foot tapping analysis

Regarding the classification based on the features extracted from both the finger and foot tapping, for each case (Case A and Case B) we derived three subcases depending on the number of features considered for the classification step. Here, we list all the analyzed subcases:

- Subcase A.1: all the 8 finger tapping features;
- Subcase A.2: all the 8 foot tapping features;
- Subcase A.3: both finger and foot tapping features.

- Subcase B.1: all the 8 finger tapping features;
- Subcase B.2: all the 8 foot tapping features;
- Subcase B.3: both finger and foot tapping features.

#### Classification evaluation metrics

Each analyzed classifier has been tested using 5-fold cross-validation and evaluated in terms of Accuracy (Eq. 1), Sensitivity (Eq. 2) and Specificity (Eq. 3), where True Positive (TP), True Negative (TN), False Positive (FP) and False Negative (FN) numbers are computed using the Confusion Matrix reported in Table 3 for a binary classifier example.

1 $$ Accuracy = \frac{TP+TN}{TP+TN+FP+FN}   $$

**Table 3:** Confusion Matrix for performance evaluation of a binary classifier

		True condition	
		*Positive*	*Negative*
Predicted condition	*Positive*	TP	FP
	*Negative*	FN	TN

2 $$ Sensitivity = \frac{TP}{TP+FN}   $$

3 $$ Specificity = \frac{TN}{TN+FP}   $$

## Results

All the participants were able to complete both clinical and instrumented evaluations. Here we report the main achievements in terms of Accuracy, Sensitivity and Specificity for the classification algorithms.

### Gait and postural analysis

We reported and compared the results obtained with both the best SVM-based and optimized ANN classifiers in Table 4. In detail, the comparison has been evaluated analyzing the average values of Accuracy, Sensitivity and Specificity across the 5-fold cross-validations.

**Table 4:** Postural and gait analysis

		Accuracy	Sensitivity	Specificity
		[%]	[%]	[%]
Subcase A.1	SVM	73.4 ±4.3	78.0 ±5.4	68.2 ±7.3
	ANN	84.7 ± 8.6	82.6 ± 14.0	86.7 ± 13.2
Subcase A.2	SVM	78.5 ±3.4	81.7 ±4.9	74.8 ±5.5
	ANN	89.4 ± 8.2	87.0 ± 12.7	91.8 ± 11.0
Subcase B.1	SVM	83.6 ±3.9	67.3 ±6.8	96.3 ±4.4
	ANN	87.9 ± 9.7	76.5 ± 21.7	97.0 ± 9.3
Subcase B.2	SVM	88.7 ±3.9	78.9 ±6.0	96.3 ±5.1
	ANN	95.0 ± 7.1	90.0 ± 15.7	99.0 ± 4.3

ANN and SVM performance comparison with all the features and selected features

**Fig. 6:**
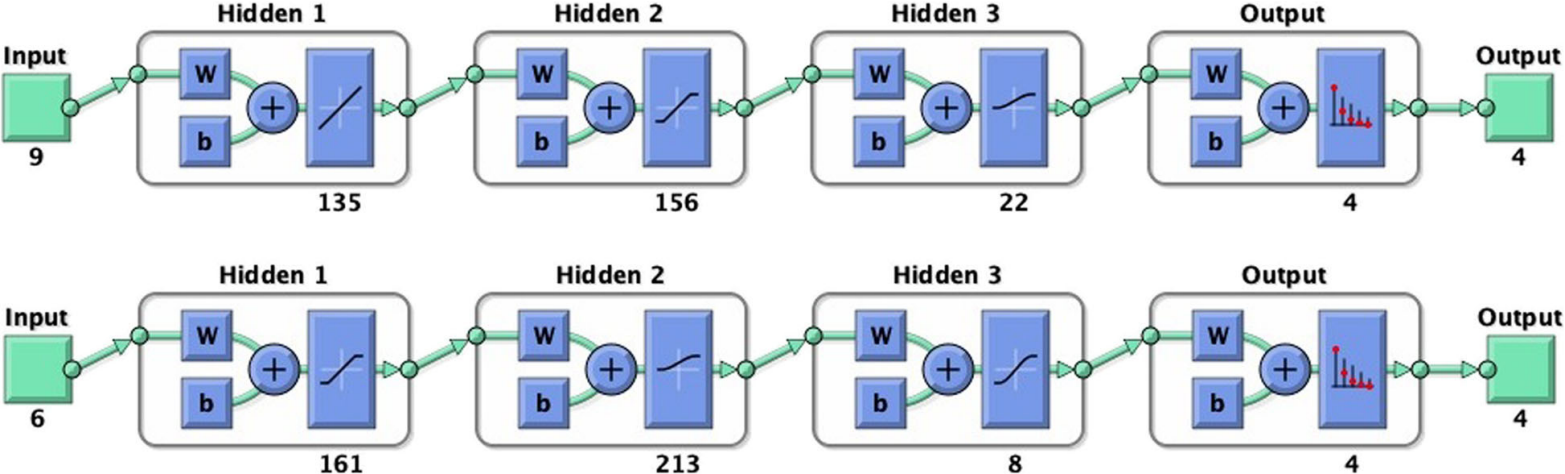
Optimal ANN topologies. Optimal topologies for ANNs obtained with the procedure based on the genetic algorithm: (top) Case A.2: Dataset with only 9 Features, (bottom) Case B.2: Dataset with only 6 Features

In Table 4 we reported the classification performance comparison between ANN and the best SVM-based classifiers for each studied subcases. The results showed that the ANN classifier performed the best for each considered subcase. In particular, when diagnosing PD (Case A), the ANN reached 89.4% (±8.2%) of Accuracy, 87.0% (±12.7%) of Sensitivity and 91.8% (±11.0%) of Specificity with only 9 selected features; while, the ANN reached 95.0% (±7.1%) of Accuracy, 90.0% (±15.7%) of Sensitivity and 99.0% (±4.3%) of Specificity with the 6 selected features in classifying mild versus moderate PD patients (Case B). The optimized topologies of the best ANN classifiers for each case are reported in Fig. 6.

### Finger tapping and foot tapping analysis

Here we reported and compared the results obtained with the best SVM for each of the three presented subcases. In detail, the comparison has been evaluated analyzing the average values of Accuracy, Sensitivity and Specificity across the 5-fold cross-validations (see Table 5).

**Table 5:** Finger tapping and foot tapping analysis

		Accuracy	Sensitivity	Specificity
		[%]	[%]	[%]
Case A	A.1	71.0±2.4	75.7±1.4	65.5±1.4
	A.2	85.5±1.7	91.0±4.2	79.0±5.2
	A.3	87.1±3.6	87.7±3.1	86.0±1.7
Case B	B.1	57.0±2.3	100.0	0.0
	B.2	81.0±1.2	84.0±1.7	78.0±2.9
	B.3	78.0±5.2	89.0±4.2	64.0±3.7

Classification indices: performance comparison among the best classifiers trained for each studied sub-case

Considering the Case A, i.e. "Healthy subjects vs PD patients" classification, we reported the results of the best trained SVM-based classifier: (sub-case A.1) the Gaussian SVM reached an accuracy of 71.0% (±2.4), a sensitivity of 75.7% (±1.4) and a specificity of 65.5% (±1.4); (sub-case A.2) the Gaussian SVM reached an accuracy of 85.5% (±1.7), a sensitivity of 91.0% (±4.2) and a specificity of 79.0% (±5.2); (sub-case A.3) the Quadratic SVM reached an accuracy of 87.1% (±3.6), a sensitivity of 87.8% (±3.1) and a specificity of 86.0% (±1.7). Also concerning the Case B, i.e. "Mild PD patients vs Moderate PD patients" classification, we reported the results of the best trained SVM-based classifier: (sub-case B.1) the Gaussian SVM reached an accuracy of 57.0% (±2.3), a sensitivity of 100% and a specificity of 0.0%; (sub-case B.2) the Gaussian SVM reached an accuracy of 81.0% (±1.2), a sensitivity of 84.0% (±1.7) and a specificity of 78.0% (±2.9); (sub-case B.3) the Gaussian SVM reached an accuracy of 78.0% (±5.2), a sensitivity of 89.0% (±4.2) and a specificity of 64.0% (±3.7).

## Discussion

In the last decade, ML techniques have been used and compared for PD classification [9]. However, most of the published studies investigate two-group classifications, i.e. PD patients vs healthy subjects, with good results obtained [10]. In this work, we have also focused on the classification between groups of patients featuring different severity levels.

The Microsoft Kinect v2 sensor has been widely utilized for PD-related research, however we noticed that most of the research studies focused on comparisons of the Kinect device with respect to optoelectronic systems. The previous studies that focus on the lower limbs usually evaluate only kinematic parameters, whereas we introduced a vision system that is able to recognize and rate PD’s motor features taking into account also postural oscillations and kinematics of upper body parts (trunk, neck and arms) while walking.

Gait and postural features have been organized by domains, and classification was carried out considering either the complete set of extracted features or a subset of them selected with a correlation based feature selection algorithm. We found out that:

- the spatial and the angular domains were the most relevant in terms of information content after feature selection phase;
- the reduced dataset for Case A (A2) showed better results in terms of classification with: 89.4% (±8.6%) of Accuracy, 87.0% (±12.7%) of Sensitivity and 91.8% (±11.1%) of Specificity;
- the reduced dataset for Case B (B2) performed the best in terms of classification with 95.0% (±7.1%) of Accuracy, 90.0% (±15.7%) of Sensitivity and 99.0% (±4.3%) of Specificity.

Our findings suggest that postural variables were the most relevant features associated with PD, which confirmed the importance of postural attitudes during walking in the neurodegeneration process. In fact, only 9 features were necessary, i.e. Double Support Phase, Stride Cadence, Stride Velocity, Swing Velocity, Stride Length, Trunk and Neck Flexion, Pisa Syndrome and Arm Swing, to diagnose PD (Case A) and only 6 features, i.e. Double Support Phase, Stride Time, Stride Length, Trunk and Neck Flexion and Arm Swing, were able to rate the severity level in PD patients (Case B).

Considering the classification based on the finger and foot tapping exercises, we first investigated the ability of the extracted features to distinguish between healthy subjects and PD patients using an SVM-based classifier. As first step, we analyzed the finger tapping (FiT) and the foot tapping (FoT) features independently, then we analyzed the features extracted from both exercises movements together. The main findings of this analysis indicate that:

- the features extracted from the foot tapping exercise lead to a better classification in terms of all the three computed indices when compared with the finger tapping: accuracy (FoT: 85.5% (±1.7) vs FiT: 71.0% (±2.4)), sensitivity (FoT: 91.0% (±4.2) vs FiT: 75.7% (±1.4)) and specificity (FoT: 79.0% (±5.2) vs FiT: 65.5% (±1.4));
- using the features extracted from both exercises (FoT and FiT) the SVM classifier performs better than the two classifiers based either on the FiT features or the FoT features. In particular, the classifier based on both feature sets reached a better accuracy (87.1% (±3.6)), a better specificity (86.0% (±1.7)) and a slight lower sensitivity (87.7% (±3.1)).

Hence, analysis of the Case A indicates that the set of features we selected from the movement acquired during both the finger and foot tapping can be used to capture the abnormal motor activity of a PD patient with great results. We also investigated the contribution of the features extracted from the finger and foot tapping exercises to distinguish between mild PD patients and moderate PD patients using an SVM-based classifier. As done for the "Healthy subjects vs PD patients" classification, we first analyzed the finger tapping (FiT) and the foot tapping (FoT) features independently, then we analyzed the features extracted from both movements together. The main findings of this analysis indicate that:

- the FiT features are not representative of the difference between mild and moderate PD subjects (accuracy 57.0% (±2.3), sensitivity 100% and specificity 0.0%);
- the FoT extracted features lead to best accuracy (81.0% (±1.2)) and specificity (78.0% (±2.9));
- the SVM classifier that use both feature sets is characterized by both a slight lower accuracy (78.0% (±5.2)) and specificity (64.0% (±3.7)) than the SVM that uses only the FoT features, and the best sensitivity level that is equal to 89.0% (±4.2).

Hence, the analysis of the Case B indicates that the set of features we selected from the movement acquired during both FoT and FiT exercises lead to a good "Mild PD patients vs Moderate PD patients" classification results, but with classification scores that are slightly lower than the "Healthy subjects vs PD patients" classification ones. It is also worth noting that the FoT features are the most important ones to achieve the best accuracy and specificity levels, and that the extracted FiT features are not representative at all of the motor differences between mild and moderate PD patients since the FiT features lead to lower accuracy and specificity levels. Only when the FiT features are used together with the FoT features the SVM classifier presents a better sensitivity level at the expense of both the accuracy and specificity.

Even though the number of tested patients is comparable to the number of PD patients involved in previous published studies [9], the major limitation of the study regards the analysis of just two levels of PD severity. Future studies might indeed consider not only mild and moderate PD patients but also slight and severe ones.

## Conclusions

Parkinson’s Disease influences a large part of worldwide population. About 1% of the population over 55 years of age is affected by this disease. Most of the current methods used for evaluating PD heavily, e.g. UPDRS scale, rely on human expertise. In this work we designed, implemented and tested a low-cost vision-based tool to automatically evaluate the motor abilities of PD patients for rating the disease severity. We investigated both motor abilities of the upper (finger and foot tapping analysis) and lower body (postural and gait analysis). Regarding the postural and gait analysis, we initially considered sixteen features: 7 temporal parameters (i.e., Stance phase %, Swing phase %, Double Support phase %, Stance time, Swing time, Strike time, and Stride cadence), 5 spatial parameters (i.e., Step length, Stride velocity, Swing velocity, Stride length, Step width), and 4 postural parameters (i.e., Average Trunk and Neck Flexion, Pisa Syndrome, Arm Swing Range of Motion). Results showed that the ANN classifier performed the best by reaching 89.4% of accuracy with only nine features in diagnosis PD and 95.0% of accuracy with only six features in rating PD severity. We found out that postural features were relevant in both cases and to our knowledge no previous studies have investigated in depth the role of these components in classification and rating of PD. Concerning the Finger and Foot tapping analysis, we extracted eight main features from the trajectories of the acquired movements using image processing techniques. Several SVM classifiers have been trained and evaluated to investigate whether the selected sets of features can be used to detect the main differences between healthy subjects and PD patients, and then between mild PD patients and moderate PD patients. Results showed that an SVM using the features extracted by both finger and foot tapping exercises is able to classify between healthy subjects and PD patients with great performances by reaching 87.1% of accuracy, 86.0% of specificity and 87.7% of sensitivity. The results of the classification of mild vs moderate PD patients indicated that the foot tapping features are the most representative ones compared to the finger tapping features. In fact, the SVM based on the foot tapping features reached the best score in terms of accuracy (81.0%) and specificity (78.0%).

From our findings, we can conclude that automatic vision system based on the Kinect v2 sensor together with the selected extracted features could represent a valid tool to support the assessment of postural, and spatio-temporal characteristics acquired from gait, finger and foot tapping in participants affected or not by the PD. In addition, the sensitivity of the Kinect v2 sensor could support medical specialists in the assessment and rating of PD patients. Finally, the low-cost cost feature and the easy and fast setup phase of the designed and implemented tool support and encourage its usability in a real clinical scenario. Feature work will focus on the integrated analysis of the data acquired during the three exercises. Moreover, a further analysis could consider a higher number of patients performing more kind of exercises. Finally, deep learning techniques might be evaluated considering the amount of big data that could be generated [66, 67].

## Data Availability

The datasets generated and/or analyzed during the current study are not publicly available due restrictions associated with anonymity of participants but are available from the corresponding author on reasonable request.

## Abbreviations

ANN: Artificial neural network
FiT: Finger tapping
FN: False negative
FoT: Foot tapping
FP: False positive
GA: Genetic algorithm
IMU: Inertial measurement units
ISW: Initial swing
LR: Loading response
ML: Machine learning
MDS-UPDRS: Movement disorder society –unified Parkinson’s Disease rating scale
MST: Mid-stance
MSW: Mid-swing
PD: Parkinson’s disease
PSW: Pre-swing
ROM: Range of motion
SVM: Support vector machine
TN: True negative
TNR: True negative rate
TP: True positive
TPR: True positive rate
TST: Terminal stance
TSW: Terminal swing
